# Predictors of disease worsening defined by progression of organ damage in diffuse systemic sclerosis: a European Scleroderma Trials and Research (EUSTAR) analysis

**DOI:** 10.1136/annrheumdis-2019-215145

**Published:** 2019-06-21

**Authors:** Mike Becker, Nicole Graf, Rafael Sauter, Yannick Allanore, John Curram, Christopher P Denton, Dinesh Khanna, Marco Matucci-Cerinic, Janethe de Oliveira Pena, Janet E Pope, Oliver Distler

**Affiliations:** 1 Department of Rheumatology and the Centre of Experimental Rheumatology, University Hospital Zurich, Zurich, Switzerland; 2 Graf Biostatistics, Winterthur, Switzerland; 3 Big Data Institute, Li Ka Shing Centre for Health Information and Discovery, Nuffield Department of Medicine, University of Oxford, Oxford, UK; 4 Rheumatology A Department, Paris Descartes University, Sorbonne Paris Cité, Cochin Hospital, Paris, France; 5 Data Science and Analytics, Bayer plc, Reading, UK; 6 UCL Division of Medicine, Royal Free Campus, London, UK; 7 Division of Rheumatology, Department of Internal Medicine, University of Michigan Scleroderma Program, University of Michigan, Ann Arbor, Michigan, USA; 8 Department of Experimental and Clinical Medicine, University of Florence, Florence, Italy; 9 Bayer US LLC, Whippany, New Jersey, USA; 10 Department of Medicine, Division of Rheumatology, University of Western Ontario, St. Joseph's Health Care, London, Ontario, Canada

**Keywords:** systemic sclerosis, disease worsening, mortality, predictive factors

## Abstract

**Objectives:**

Mortality and worsening of organ function are desirable endpoints for clinical trials in systemic sclerosis (SSc). The aim of this study was to identify factors that allow enrichment of patients with these endpoints, in a population of patients from the European Scleroderma Trials and Research group database.

**Methods:**

Inclusion criteria were diagnosis of diffuse SSc and follow-up over 12±3 months. Disease worsening/organ progression was fulfilled if any of the following events occurred: new renal crisis; decrease of lung or heart function; new echocardiography-suspected pulmonary hypertension or death. In total, 42 clinical parameters were chosen as predictors for the analysis by using (1) imputation of missing data on the basis of multivariate imputation and (2) least absolute shrinkage and selection operator regression.

**Results:**

Of 1451 patients meeting the inclusion criteria, 706 had complete data on outcome parameters and were included in the analysis. Of the 42 outcome predictors, eight remained in the final regression model. There was substantial evidence for a strong association between disease progression and age, active digital ulcer (DU), lung fibrosis, muscle weakness and elevated C-reactive protein (CRP) level. Active DU, CRP elevation, lung fibrosis and muscle weakness were also associated with a significantly shorter time to disease progression. A bootstrap validation step with 10 000 repetitions successfully validated the model.

**Conclusions:**

The use of the predictive factors presented here could enable cohort enrichment with patients at risk for overall disease worsening in SSc clinical trials.

**Key messages**

**What is already known about this subject?**

- Capturing the complexity and heterogeneity of systemic sclerosis in clinical trials is difficult.
- The widely used modified Rodnan skin score failed in recent clinical trials as a surrogate parameter for universal disease progression. Using worsening of organ involvement as a study endpoint is impeded by its relative sparsity.

**What does this study add?**

- We have identified factors that are associated with disease progression and lead to organ failure.

**How might this impact on clinical practice or future developments?**

- The factors identified here could be used to select patients at risk of progressive organ involvement for clinical trials.
- Identifying patients at risk also has implications for clinical care.

## Introduction

Systemic sclerosis (SSc), a rheumatic disease characterised by autoimmunity, tissue fibrosis and vasculopathy, has a high mortality rate compared with other rheumatic diseases.^1 2^ Mortality in SSc is the result of organ involvement, with lung disease (either interstitial lung disease or pulmonary arterial hypertension (PAH)) being the most prominent risk factor for death.^3^ Skin fibrosis is a hallmark of SSc and is easily measurable in a standardised manner using the modified Rodnan skin score (mRSS), which has good inter-rater and intrarater variability.^4–7^ The mRSS also correlates with organ involvement; the rate of progression of skin thickness can predict early mortality in patients with diffuse SSc (dcSSc), while early worsening of mRSS (within 12 months) is associated with poorer survival and increased disease progression^8 9^ Consequently, the mRSS is widely used as a primary outcome parameter in clinical trials of patients with dcSSc or as a part of composite indices.^9–12^ However, skin fibrosis is only a surrogate marker for overall disease progression,^13^ and the use of more relevant endpoints, such as worsening organ function or death, is desirable for clinical trials and is increasingly requested by regulatory authorities. One methodological limitation is that these events are relatively rare in unselected patients. In clinical trials of dcSSc, enriching a dataset with these endpoints requires identification of predictive factors associated with organ worsening or death; these factors can then be considered as inclusion criteria for clinical trial designs with enriched patient populations. We aimed to identify predictive factors for disease worsening and death in patients with dcSSc by analysing data from the large European Scleroderma Trials and Research (EUSTAR) group database.

## Methods

### Patients and study design

This study used prospectively collected data from the EUSTAR database. The structure of the EUSTAR database and minimum essential dataset have been described previously.^14 15^

Data from patients with dcSSc (as defined by LeRoy *et al*)^16^ were included if they had a visit in 2009 or later (defined as the baseline visit) and either a follow-up visit or death within 12±3 months after baseline. Twelve months was chosen as the primary analysis point, as this reflects the usual study duration of SSc trials targeting fibrosis.

### Definition of disease worsening

An expert group (YA, MM-C, CPD, OD, JP) defined the combined endpoint of disease worsening, which was agreed on by nominal group technique. A patient was considered to have organ worsening if he or she fulfilled any of the following criteria within 12±3 months of the baseline visit: new-onset renal crisis; decrease in forced vital capacity (FVC)≥10%; new left ventricular ejection fraction (LVEF)<45% or decrease in LVEF by>10% for patients with baseline LVEF<45%; new-onset echocardiography-suspected pulmonary hypertension (PH) (as defined by the treating physician); or death. Variable definitions, recoding of variables and handling of missing values are described in the online supplementary appendix.

10.1136/annrheumdis-2019-215145.supp1 Supplementary data

### Statistical analysis

For variables with >10% missing data, the missing not at random (MNAR) assumption was explored where possible, but the analyses did not support the assumption of MNAR, instead random missingness was assumed (see online supplementary appendix for more details).

Missing data were imputed on the basis of multiple imputation (MI) using the R package *mice*. For the imputation model, all 42 variables from the full model, including the dependent variable ‘disease worsening’, were included. With the function *quickpred*, predictors were automatically selected (1) with an absolute correlation with the target variable of ≥0.2 and (2) with a proportion of usable cases (ie, cases with missing data on the target variable that had observed values on the predictor) of ≥0.25. The order of variable imputation was defined according to the number of missing cases. Depending on the scale of the target variable, MI was performed using either linear regression (*norm.nob*), logistic regression (*logreg*) or polytomous, ordered regression (*polr*) for factors with more than two levels. Based on the fraction of missing information,^17^ 100 imputed datasets were generated. Further details of statistical analysis are described in the online supplementary appendix.

### Patient and public involvement

This was a retrospective study using a registry with patient data from different primary investigation sites. However, neither direct patients nor the public were involved. Study results will be disseminated within patient communities via the Federation of European Scleroderma Associations and its patient congresses.

## Results

### Baseline characteristics

In total, 1451 patients met the inclusion criteria at the time of data extraction (10 February 2016). Of these, 706 had data on the presence of the combined endpoints available and were included in the analysis. Patient baseline characteristics are shown in table 1, with a comparison between the 706 included patients and the 745 excluded patients shown in the online supplementary table S2. There was no major difference between these groups, although numerically, patients without missing data had a slightly higher disease duration and more renal crises but less frequent active disease. Hence, there was no major selection bias. However, a bias based on unmeasured variables cannot be excluded.

**Table 1:** Clinical and demographic characteristics of the 706 patients from the EUSTAR database included in the analysis

Characteristics	Patients (n=706)	Available data (% patients)
*Demographic*		
Male sex	172 (24.4)	100
Age, mean±SD	52.9±12.9	100
Disease duration, months (mean±SD)	101.1±94.0	94.1
Body weight, kg (mean±SD)	64.6±13.4	97.2
*Laboratory parameters*		
ANA positive	657 (94.4)	98.6
ACA positive	48 (7.1)	96.3
Anti-Scl70 positive	414 (60.2)	97.5
Anti-U1RNP positive	27 (4.7)	81.3
Creatine kinase elevation	64 (9.5)	95.2
Proteinuria	57 (8.4)	95.6
Hypocomplementaemia	39 (6.3)	88.1
ESR>20 mm/1 hour, mean±SD	25.3±20.6	94.5
CRP elevation	190 (27.7)	97.0
*Vascular*		
Raynaud’s present	683 (96.7)	100
DU ever	266 (38.1)	98.9
Active DU*	126 (18.1)	98.7
Scleroderma (puffy fingers)	303 (44.2)	97.2
Worsening of finger vascularisation within the last month	162 (23.3)	98.3
*Musculoskeletal*		
Tendon friction rubs	89 (12.8)	98.3
Joint synovitis	108 (15.4)	99.3
Joint contractures	310 (44.4)	98.9
Muscle weakness	164 (23.4)	99.3
*Skin*		
mRSS, mean±SD	14.2±9.1	93.2
Worsening of skin changes within the last month	141 (20.3)	98.3
Skin progression rate, mean±SD	0.6±1.7	88.2
*Cardiopulmonary*		
Arterial hypertension	154 (21.9)	99.6
Pericardial effusion	58 (8.9)	92.5
Echocardiography-suspected PH	113 (16.3)	98.0
Conduction blocks	104 (15.6)	94.2
Abnormal diastolic function	170 (25.0)	96.2
Lung fibrosis†	131 (19.7)	94.3
Significant dyspnoea	91 (13.2)	97.7
DLCO, %predicted (mean±SD)	64.1±20.2	94.1
FVC, %predicted (mean±SD)	86.4±21.3	96.5
FEV_1_, %predicted (mean±SD)	85.0±18.7	78.3
TLC, %predicted (mean±SD)	84.2±19.9	66.1
LVEF, %predicted (mean±SD)	61.7±7.0	96.5
*Gastrointestinal*		
Oesophageal symptoms	455 (64.5)	99.9
Stomach symptoms	192 (27.4)	99.3
Intestinal symptoms	177 (25.2)	99.3
Kidney		
Renal crisis	34 (4.8)	99.4
*Disease activity*		
Active disease‡	191 (30.7)	88.1

Data are n (%) unless otherwise stated. (Percentages with characteristics were calculated from numbers of patients with data available).

Clinical manifestations were defined according to the EUSTAR definitions.^15^

Presence of significant dyspnoea was based on the judgement of the treating physician.

*Active DUs was a composite endpoint that was considered positive if either DU (from the minimal essential dataset) or digital gangrene was present.

†Lung fibrosis was defined as FVC<60% or FVC<70% and presence of lung fibrosis on high-resolution computed tomography.

‡Active disease was defined as score >3 calculated according to the EScSG disease activity indices for SSc.^38^

ACA, anti-centromere antibody; ANA, anti-nuclear antibody; CRP, C-reactive protein; DLCO, diffusion capacity of the lung for carbon monoxide; DU, digital ulcer; ESR, erythrocyte sedimentation rate;EScSG, European Scleroderma Study Group; EUSTAR, European Scleroderma Trials and Research; FEV_1_, forced expiratory volume after 1 s; FVC, forced vital capacity; LVEF, left ventricular ejection fraction;mRSS, modified Rodnan skin score; PH, pulmonary hypertension; TLC, total lung capacity.

### Predictive factors for disease worsening

Of 706 patients with available data, 228 (32.3%) fulfilled the pre-defined criteria for disease worsening within 12±3 months of the baseline visit. The most common forms of disease worsening were deterioration of FVC and death (table 2). Renal crisis and worsening of LVEF were rare.

**Table 2:** Frequency of disease worsening

Disease worsening	Yes	No	Missing
Any*	228 (32.3)	478 (67.7)	—
Worsening FVC	103 (14.6)	514 (72.8)	89 (12.6)
Death within 12 (±3) months	92 (13.0)	614 (87)	
New echocardiography-suspected PH	37 (5.2)	582 (82.4)	87 (12.3)
New renal crisis	7 (1.0)	613 (86.8)	86 (12.2)
Worsening LVEF	5 (0.7)	614 (87.0)	87 (12.3)

Data are n (%).

*Patients were considered to have disease worsening if death occurred within 12±3 months after baseline or if worsening was present for any of the other components.

FVC, forced vital capacity; LVEF, left ventricular ejection fraction; PH, pulmonary hypertension.

**Figure 1:**
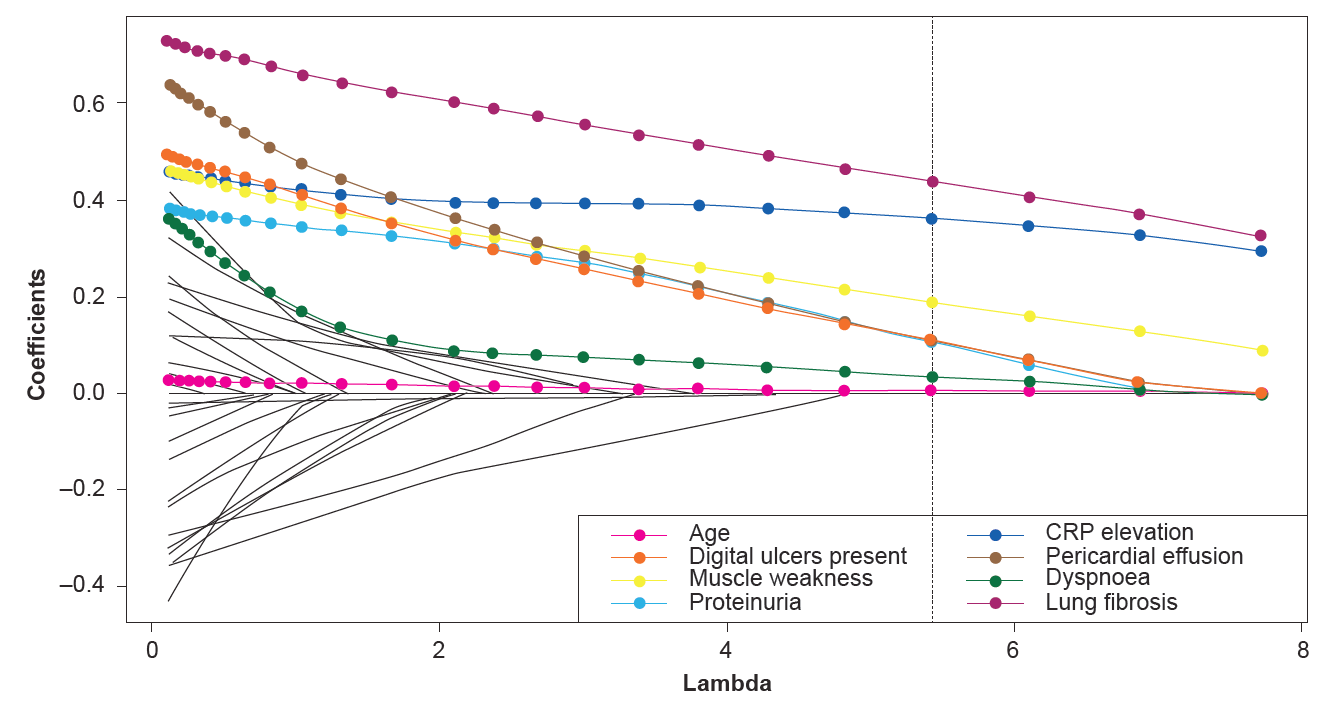
Average regression coefficients across 100 imputations plotted against the penalisation parameter, lambda. The vertical dashed line represents the selected model chosen as it had the smallest Bayesian information criterion. Traces in colour are those of the regression coefficients (and hence predictor variables) that remained in the final model. Traces for excluded regression coefficients are plotted in black and are not specified in the legend. CRP, C-reactive protein.

Figure 1 presents the multiple imputation—least absolute shrinkage and selection operator (MI-LASSO) regression coefficients based on averages across 100 imputed datasets, presented on a logarithmic OR scale evaluated for 24 varying penalisation factors (lambda). The regression coefficient estimates at each selected penalisation factor indicate the extent to which they contribute to a change in the probability of disease progression in terms of ORs in comparison to the population mean. The smaller the lambda, the larger the penalisation; therefore, average regression coefficients are shrunk towards zero. The model with the smallest Bayesian Information Criterion (BIC) was chosen as the final model. If a regression coefficient was shrunk to zero, the predictor variable was no longer retained in the final model.

Table 3 shows the final model, with OR and 95% CI based on all 100 imputed datasets. All ORs were>1 and, therefore, positively associated with disease worsening. There was substantial evidence for a strong association between disease progression and age, active digital ulcers (DUs), C-reactive protein (CRP) elevation, lung fibrosis and muscle weakness. The p values for pericardial effusion, proteinuria and significant dyspnoea suggest only weak or very weak evidence for an association with disease worsening.

**Table 3:** Final regression model for disease worsening

	p Value	OR	95% CI
**Age (years**)	**0.001**	**1.02**	**1.01 to** **1.04**
**Lung fibrosis**	**0.0004**	**2.21**	**1.43 to** **3.41**
**CRP elevation**	**0.002**	**1.80**	**1.23 to** **2.63**
**Muscle weakness**	**0.015**	**1.64**	**1.10 to** **2.45**
**Active DU**	**0.026**	**1.64**	**1.06 to** **2.54**
Proteinuria	0.064	1.75	0.97 to 3.16
Pericardial effusion	0.098	1.65	0.91 to 2.97
Significant dyspnoea	0.491	1.20	0.72 to 2.00

Parameters in **bold** had strong evidence for a significant association with disease progression in the final model.

CRP, C-reactive protein; DU, digital ulcer.

As muscle weakness was a non-objectively defined, patient-reported parameter, we aimed to further characterise patients with this symptom. In patients defined as having muscle weakness, the frequency of creatine kinase (CK) elevation was higher than in those without weakness (19.9% (n=31/156) vs 6.2% (n=32/513); p<0.0001 by χ^2^ test); a higher frequency of gastrointestinal symptoms (39.0% (64/164) vs 21.1% (113/535); p<0.0001 by χ^2^ test); and a higher number of deaths (25.0% (41/164) vs 8.9% (48/537); p<0.0001 by χ^2^ test) were also observed in those with muscle weakness compared with those without.

### Applicability and feasibility of the predictors retained in the final model

To illustrate the impact of the final model on the probability of increasing the number of patients with worsening organ function in a given selection of patients, we calculated the outcome probabilities for combinations of risk factors from the final model in the 706 study patients (table 4). As suggested by the high ORs for lung fibrosis and CRP elevation (see table 3), these two factors alone increased the probability for an event during the observation time to 52.0% in patients aged 60 years and 57.9% in patients aged 70 years (table 4) compared with 32.2% for the overall study population. If patients had lung fibrosis, muscle weakness, DU and CRP elevation, the probability for an event was 74.5% at 60 years and 78.8% at 70 years (table 4). However, depending on the number of predictors included, the number of selected patients decreased, for example, the optimal combination for maximum enrichment left only eight patients who had lung fibrosis, muscle weakness, CRP elevation and present DUs in our study population (table 4).

**Table 4:** Probability (%) of disease worsening for combinations of predictors in study population (n=706)

Other risk factors*	Age			Patient numbers†
	60 years	65 years	70 years	
Lung fibrosis	37.5	40.4	43.3	131/666
Lung fibrosis and CRP elevation	52.0	55.0	57.9	47/650
Active DU	30.9	33.5	36.1	126/697
Lung fibrosis and active DU	49.7	52.6	55.6	31/662
Muscle weakness	30.9	33.5	36.2	164/701
Lung fibrosis, muscle weakness and active DU	61.8	64.6	67.3	16/660
Lung fibrosis, muscle weakness, CRP elevation and active DU	74.5	76.7	78.8	8/646

*Predictors not specified in each row are set to zero.

†Patient numbers irrespective of age that fulfil the criteria within the whole study population.

CRP, C-reactive protein; DU, digital ulcer.

### Impact of predictors from the final model during long-term observation

**Figure 2:**
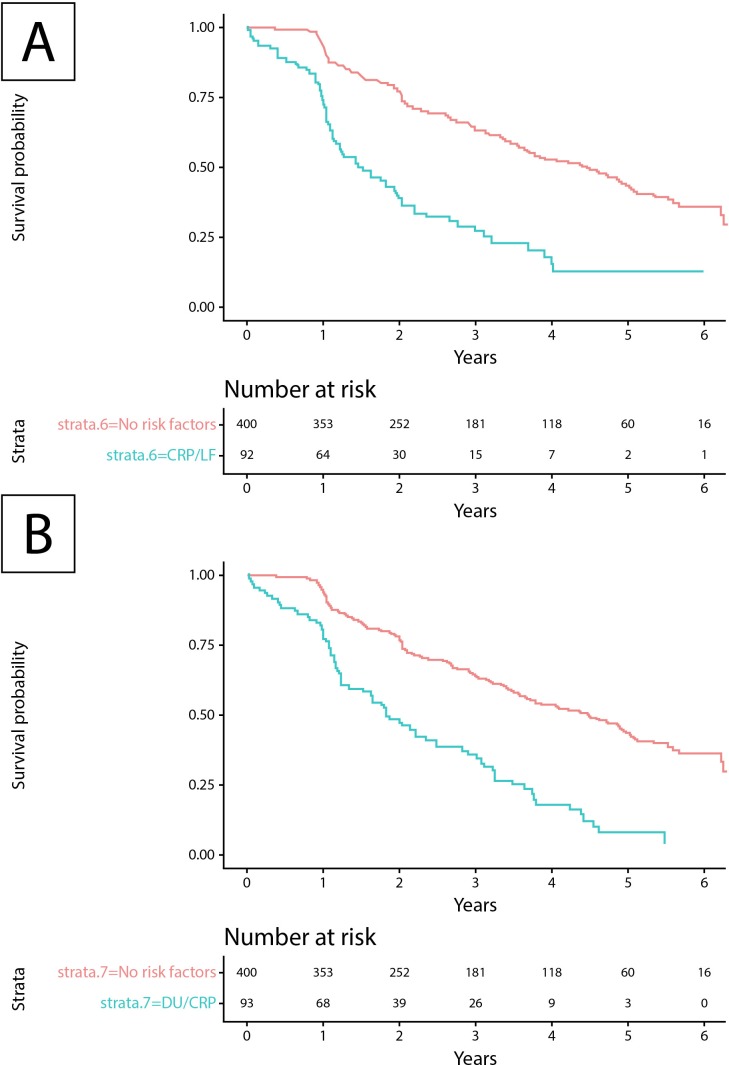
Event-free survival in patients with SSc depending on risk factors for progression of organ damage. (A) Event-free survival of patients with SSc fulfilling the inclusion criteria (diffuse SSc, death or at least one follow-up visit earliest at 12±3 months after baseline visit in 2009 or later) with risk factors (elevated CRP and presence of lung fibrosis) versus no risk factors (active DU, CRP elevation, significant dyspnoea, lung fibrosis, muscle weakness, pericardial effusion and proteinuria). The median survival time for patients with and without risk factors was 1.53 years (95% CI 1.13 to 1.99) and 4.48 years (95% CI 3.70 to 4.97), respectively. The log-rank test was significant (p<0.001). (B) Event-free survival of patients with SSc fulfilling the inclusion criteria with risk factors (elevated CRP and active DU) versus no risk factors. The median survival time for patients with and without risk factors was 1.82 years (95% CI 1.23 to 2.47) and 4.48 (95% CI 3.70 to 4.97) years, respectively. The log-rank test was significant (p<0.001). CRP, C-reactive protein; DU, digital ulcer; LF, lung fibrosis; SSc, systemic sclerosis.

To evaluate the impact of the predictors retained in the final model on survival, we additionally calculated long-term event-free survival curves for patients with SSc with and without risk factors. Specifically, we tested the most clinically feasible combinations of increased CRP and presence of lung fibrosis or DU. These combinations showed a significantly worse event-free survival with the risk factors present (figure 2). With the presence of lung fibrosis and elevated CRP, the median time to an outcome event was 1.53 years versus 4.48 years for patients without any risk factors, that is, active DU, CRP elevation, significant dyspnoea, lung fibrosis, muscle weakness, pericardial effusion or proteinuria (figure 2A; p<0.001 by log-rank test). Active DU and elevated CRP shortened the median time to an outcome event from 4.48 years to 1.82 years (figure 2B; p<0.001 by log-rank test). The additional analysis of four risk factors on their own showed that each (active DU, raised CRP, presence of lung fibrosis and muscle weakness) was associated with a significantly increased incidence of outcome events during follow-up as well as in combination (each p<0.001 by log-rank test; see online supplementary figure S5).

### Model validation

A bootstrap with 10 000 repetitions was used to validate the final model chosen by the BIC. The C-index, which is identical to the area under the receiver operating characteristic, is a good measure to estimate discrimination. This in turn refers to the ability of the model to separate patients with and without the outcome event. The final model had a C-index of 0.711, which was 0.705 at validation, indicating good calibration (ie, agreement between actual and predicted probabilities) (see online supplementary figure S1).

## Discussion

By using a novel statistical approach to analyse data from a clinical registry, we successfully identified predictors of severe disease worsening—defined as organ failure within a period of 12±3 months—in patients with dcSSc. Based on our logistic regression model, we showed that the probability of a 60-year-old patient with lung fibrosis, DU, muscle weakness and CRP elevation developing disease worsening within the observation period increases to 74.5% compared with 32.2% for the whole study population. The predictive factors of age, presence of DU, lung fibrosis, CRP elevation and muscle weakness represent important aspects of the disease and also correspond to the key characteristic features of vasculopathy (DU), autoimmunity/inflammation (CRP elevation) and tissue fibrosis (lung fibrosis). In addition to being predictive in our model for progression of disease after 12±3 months, the presence of DU, lung fibrosis, CRP elevation and muscle weakness could also predict, alone or in combination, disease progression over a longer period of time (up to 6 years after the baseline visit). This confirms the role of CRP elevation as an indicator of active disease and its potential relevance as an inclusion criterion in trials.^18–21^ The data also support the notion that the presence of muscle weakness may include patients with overt myositis/myopathy, as well as patients with gastrointestinal problems being more likely to have malnutrition and consecutive muscle weakness.

DUs were identified in a previous EUSTAR study as a risk factor for cardiovascular worsening and mortality.^22^ Lung involvement in SSc is well known,^3^ and this is reflected in the high incidence of worsening FVC (14.6%) in the present study, while development of PH was the third most frequent event (5.2%) leading to disease worsening. The relatively high frequency of the FVC endpoint corresponds to the high OR for lung fibrosis in the final model. The discriminative value of a 10% decline in FVC has recently been confirmed by another report showing that this magnitude of decline is associated with increased mortality.^23^ The percentage of patients with a significant FVC decline is similar to the patients with SSc receiving placebo from the Scleroderma Lung Study I/II analysis (approximately 15%).^24^ The percentage of patients developing new echocardiography-suspected PH in our analysis was slightly higher than in the at-risk population included in the Pulmonary Hypertension Assessment and Recognition of Outcomes in Scleroderma registry (7% at 2 years)^25^ and much higher than in unselected patients, where the annual incidence is approximately 1.4%–1.5%.^26 27^ This is most likely due to a combination of patient selection in our cohort and use of an echocardiography-suspected PH definition that was not strictly based on right heart catheterisation data but based on assessment by the treating physician. The mortality rate within 12±3 months among patients with dcSSc in the present study (13%) was relatively high compared with earlier reports from the EUSTAR database (5-year mortality from diagnosis in all patients with SSc of 11%)^28^ but also surpasses the 13% early mortality (within 3 years) recently reported in a multinational inception cohort.^29^ However, the cohort used in the present analysis was selected to include dcSSc only, and thus was prone to have more complications than a general SSc cohort. In addition, deaths in the present study were all-cause deaths and not limited to SSc-related causes, with 63.3% of deaths documented as SSc related. However, currently, there is no clear definition of SSc-related mortality.

Our study was designed to address two important limitations that are often encountered when searching for predictive factors in large datasets from patient registries. First, there is often a high incidence of missing data, and second there are limits to the number of potential predictive variables that can be included in the statistical model. Observations with missing data on any predictor variable will be eliminated in the process of ordinary logistic regression, so that only a ‘full dataset’ with valid data on all candidate predictor variables can be used in the final analysis. As patient registries such as EUSTAR depend on data input from many clinical centres, they typically have a certain amount of missing data. In addition, a low incidence of outcome events limits the number of predictive variables that can be used for the analysis, as the ratio of outcome events to predictor variables in the model should ideally be 1:10 or lower.^30^ The issue of missing data was addressed in our analysis by imputing missing data on the basis of MI. The issue of limited predictive variables was addressed by using LASSO, a different type of regression analysis that allows selection and reduction of predictor variables (‘shrinkage’).^31^

It is possible that collection of some variables in the EUSTAR database began only recently or changed their definition during the data collection period. For example, PH is now mainly recorded as PAH. While PAH is currently defined in EUSTAR by mean pulmonary arterial and wedge pressures measured during right heart catheterisation, when the registry was initiated, PH was estimated by echocardiography. Hence, echocardiography-suspected PH in our study possibly overestimates true PAH. It seems likely that, as genuine PAH is strongly associated with mortality in SSc, the 92 deaths (13%) include some deaths resulting from PAH. Despite these limitations, our novel approach to the data in the EUSTAR registry successfully identified clinical features that allow enrichment of patients with dcSSc with disease worsening defined by organ failure.

Enriching a recruitment for clinical trials for progression of organ damage is not the same as for disease worsening as defined by progression of skin fibrosis. Therefore, the predictive factors that allow selection of patients with a higher probability for future increase in mRSS—baseline mRSS, joint synovitis, age, gender, disease duration and CK elevation^32^—are different. mRSS is a validated marker of overall disease severity and progression; baseline mRSS predicts both worsening and improvement of skin fibrosis,^33^ progression of skin fibrosis within 1 year is associated with a decline in lung function and decreased survival, and skin progression rate and trajectories are linked to increased mortality and the risk of renal crisis.^12 13 34^ However, skin fibrosis remains a surrogate marker and is not a direct measure of overall disease morbidity and mortality. In addition, it did not perform well as a primary outcome measure in recent randomised controlled trials for SSc^35–37^ (while other secondary and exploratory endpoints such as FVC and patient-reported outcomes have shown promising trends), indicating that the mRSS has inefficient sensitivity to change according to morbidity.^18^

Clinical trial design in SSc is undergoing major changes in the selection of endpoints; it is likely that these will change from use of mRSS as the most common primary endpoint to other items or indices considering progression of organ involvement, overall disease progression and death. In addition to the endpoints used in this study, these could also encompass the mRSS or other outcomes including gastrointestinal involvement (weight loss), digital ischaemia, myopathy, disability and other features that have an impact on patients’ lives. So far, little information has been available about which patient cohorts could be used for these analyses to allow for enough events and to make these novel study designs possible. This study provides evidence-based information from the largest SSc database available worldwide regarding which patients are appropriate for inclusion in these clinical trials. Although self-reported muscle weakness is difficult to use in clinical trials, an increased CRP and the presence of lung fibrosis and DUs are feasible inclusion criteria for further clinical trials. However, the selection of enrichment criteria for a clinical study must be balanced against feasibility of recruitment and representation of a broader SSc population. Hence, this study provides key data to inform a novel study design that could likely be applied in the near future.
