# Supplementary material for: Predictors of disease worsening defined by progression of organ damage in diffuse systemic sclerosis: a European Scleroderma Trials and Research (EUSTAR) analysis
Source: Ann Rheum Dis. 2019 Jun 21;78(9):1242–8. doi: 10.1136/annrheumdis-2019-215145 (PMC6788922; doi:10.1136/annrheumdis-2019-215145)
Supplement: Supplementary data [file annrheumdis-2019-215145supp001.pdf]

SUPPLEMENTARY APPENDIX

Predictors for disease worsening defined by organ failure in diffuse systemic sclerosis: a European scleroderma trials and research (EUSTAR) analysis

Mike O Becker[1], Nicole T Graf[2], Rafael Sauter[3], Yannick Allanore[4], John Curram[5], Christopher P Denton[6], Dinesh Khanna[7], Marco Matucci-Cerinic[8], Janethe de Oliveira Pena[9], Janet E Pope[10], Oliver Distler[1]

STATISTICAL ANALYSIS

Selection and elimination of predictor variables ('shrinkage') was conducted using the least absolute shrinkage and selection operator (LASSO) method, a penalised regression approach that performs variable selection through regularisation. The applied LASSO regression was adapted to account for multiple imputation (MI) such that the model selection yielded consistent results, which is not guaranteed if standard LASSO is repeatedly applied to several imputed datasets.(21, 35-37) The MI-LASSO approach is a type of group LASSO regression (38) that has superior model-choice properties compared with stepwise model selection strategies or LASSO regression including complete cases only.(21)

Predictors without face validity and not clinically useful (as judged by the clinical experts) as well as predictors with ≥50% missing values were excluded from the regression model. Two variables with ~40% missing cases were also excluded to achieve a proportion of ≥25% complete cases (online supplementary table S1), increasing the accuracy of the MI-LASSO analysis. This resulted in 42 remaining predictors (online supplementary table S1). Most of the predictors had few missing cases.

Becker M, *et al. Ann Rheum Dis* 2019; 0:1242–1248. doi: 10.1136/annrheumdis-2019-215145

The optimal shrinkage parameter based on the MI-LASSO analysis and the corresponding model with regression coefficients was determined according to the optimal Bayesian information criterion (BIC) model choice criteria as suggested by Chen and Wang (2013) (21).

Bootstrap with 10 000 repetitions was used to validate the logistic model. Missing values were imputed with single conditional mean imputation, as validation of a model with MI data is not possible.

All analyses were performed using SPSS version 20 or R 3.3.32.5 (packages Hmisc, rms, mice, and survival).

VARIABLE DEFINITIONS, RECODING AND HANDLING OF OUTLIERS

Impossible values for disease duration (<0 years) and for body mass index (BMI) (>70 kg/m^2) were set to missing.

The categories of scleroderma (puffy fingers) were recoded as follows: 0=No / Never; 1= Yes / Current / Previously.

Pericardial effusion was recoded as a binary variable (0=0; >0=1).

Modified skin progression rate was calculated as the ratio of modified Rodnan skin score (mRSS) at baseline to disease duration in months.

Lung fibrosis was defined as fulfilling either of two conditions:

- Forced vital capacity (FVC) <60%
- FVC <70% and presence of lung fibrosis on high-resolution computed tomography

Valentini activity index (VAI) was calculated by totalling the scores of the ten Valentini index items (online supplementary table S3). Patients who scored >3 with the

Becker M, *et al. Ann Rheum Dis* 2019; 0:1242–1248. doi: 10.1136/annrheumdis-2019-215145

available items were defined as active. Patients who did not score >3 with the available items, and for whom the missing variables (if positive) would not add up to >3, were defined as inactive. Patients who did not score >3, but for whom the missing items (if positive) could change the activity score to active, were defined as unknown.

mRSS was defined with help of the variable ‘modified Rodnan skin score only imported value’. If this variable had missing values, a sum score of all 17 body parts (‘face’ to ‘left foot’) was calculated, provided that there were at least six valid values including valid values for left and right fingers.

Pulmonary hypertension was defined as present or not present based on assessment of the echocardiogram by the treating physician/cardiologist and was not explicitly based on the systolic pulmonary arterial pressure (measured by echocardiography) or right heart catheterisation values.

Dyspnoea was considered to be significant based on the judgement of the treating physician.

Presence of digital ulcer (DU) was a composite endpoint that was considered positive if either DU (from the minimal essential dataset) or gangrene was present (gangrene was present only in a minority of patients).

Death was all-cause mortality. If the treating physician deems a death SSc-related, there is a box that can be ticked in the database. According to this information, 50/79 deaths (63.3%) that occurred were due to SSc, 29/79 deaths were not due to SSc (36.7%) and for 10/79 deaths (12.7%) there was no information on this.

Becker M, *et al. Ann Rheum Dis* 2019; 0:1242–1248. doi: 10.1136/annrheumdis-2019-215145

CHOOSING PREDICTOR VARIABLES

Patients could be included in the analysis even if they had a prior occurrence of one of the composite endpoints (excluding death), as further progression of disease worsening was still possible.

Selection of predictors focused on clinically relevant variables and those with face validity. In addition, predictors with ≥50% missing values were not considered for inclusion; thus, 44 predictors with <50% missing values for the analysis remained.

As the accuracy of the multiple imputation – least absolute shrinkage and selection operator (MI-LASSO) analysis – decreases as the proportion of incomplete cases increases, the proportion of complete cases was set to ≥25%. To achieve a proportion of complete cases ≥25%, the two predictors with the largest amount of missing data (BMI and anti-RNA-polymerase III positive) were not considered for MI-LASSO, and thus, 42 predictors were retained.

MISSINGNESS AT RANDOM

For variables with >10% missing data (total lung capacity [TLC], forced expiratory volume after 1 second [FEV_1], anti-U1RNP positive, hypocomplementaemia, VAI and skin progression rate), an assumption of missingness not at random (MNAR) was explored. TLC correlated best with FEV_1 (online supplementary figure S2). Missingness for TLC was not associated with values for FEV_1 and vice versa, indicating that MNAR is not a likely assumption for TLC or FEV_1 (see online supplementary figure S3 and S4). Anti-U1RNP positive, hypocomplementaemia, VAI and skin progression rate did not correlate well with any of the other variables. Thus, the MNAR assumption could not be explored for these variables.

Becker M, *et al. Ann Rheum Dis* 2019; 0:1242–1248. doi: 10.1136/annrheumdis-2019-215145

NUMBER OF IMPUTATIONS

The MI-LASSO with 100 imputed datasets was applied. It was noted that the number of imputed datasets (M) had an effect on the MI-LASSO results. This was probably related to the threshold, which is used to set regression coefficients to a non-zero value.[1] The method of Chen and Wang (2013) was followed and the threshold was set to delta=10e–10.[1] The applied stop criterion depended on delta and M, and Chen and Wang (2013) presented results for M=5 imputed datasets only. It was observed that trace plots look different if M is increased, mainly because lambda is rescaled. However, the threshold may also affect the regression coefficients, which were included in the optimal model. A decision was made to redefine the stop criterion in an ad hoc way. After running the MI-LASSO, only regression coefficients with a value of >0.006 at the optimal model (according to the BIC) were included. This adapted rule for the inclusion of regression coefficients led to the same model (same selected regression coefficients) for M=5 and M=100.

IMPACT OF PREDICTORS FROM THE FINAL MODEL DURING LONG-TERM OBSERVATION

In order to evaluate the impact of the predictors retained in the final model on survival, we additionally calculated long-term event-free survival curves for patients with systemic sclerosis (SSc) with and without risk factors. Patients with diffuse cutaneous disease, for whom outcome data were available and with a baseline visit in 2009 or later and death or at least one follow-up visit earliest at 12±3 months were included. For this analysis, there was no imputation of missing values, but longer observation periods were possible. ‘No risk factors’ means that none of the following factors was present at baseline: active DU, C-reactive protein (CRP) elevation, significant dyspnoea, lung fibrosis, muscle weakness, pericardial

Becker M, *et al. Ann Rheum Dis* 2019; 0:1242–1248. doi: 10.1136/annrheumdis-2019-215145

effusion and proteinuria. Active DU as risk factor, for example, means that active DU was present at baseline, which does not preclude the presence of other risk factors. The online supplementary figures S5A to S5E show the cumulative survival curves, and online supplementary table S4 gives the corresponding median survival times for some risk factors of the final model and their combination(s).

REFERENCE

1. Chen Q, Wang S. Variable selection for multiply-imputed data with application to dioxin exposure study. Stat Med 2013;32:3646–59.

Becker M, *et al. Ann Rheum Dis* 2019; 0:1242–1248. doi: 10.1136/annrheumdis-2019-215145

SUPPLEMENTARY FIGURES AND TABLES

Supplementary table S1 List of predictors with <50% missing values and percentage of available data for included patients with valid data for the primary endpoint (n=706)

**Predictor**	**Available data (%)**
Demographic	
Sex	100.0
Age, years	100.0
Disease duration, months	94.1
Body weight, kg	97.2
BMI, kg/m^2	61.3
Laboratory parameters	
ANA positive	98.6
ACA positive	96.3
Anti-Scl70 positive	97.5
Anti-U1RNP positive	81.3
Anti-RNA-polymerase III positive	59.6
Creatine kinase elevation	95.2
Proteinuria	95.6
Hypocomplementaemia	88.1
ESR >20 mm/1h	94.5
CRP elevation	97.0
Vascular	
Raynaud's present	100.0

Becker M, *et al. Ann Rheum Dis* 2019; 0:1242–1248. doi: 10.1136/annrheumdis-2019-215145

DU	98.9
Active DU	98.7
Scleroderma (puffy fingers)	97.2
Musculoskeletal	
Tendon friction rubs	98.3
Joint synovitis	99.3
Joint contractures	98.9
Muscle weakness	99.3
Cardiopulmonary	
Pericardial effusion	92.5
Pulmonary hypertension	98.0
Conduction blocks	94.2
Abnormal diastolic function	96.2
Lung fibrosis	94.3
Arterial hypertension	99.6
Dyspnoea	97.7
DLCO, %predicted	94.1
FVC, %predicted	96.5
FEV_1, %predicted	78.3
TLC, %predicted	66.1
LVEF, %predicted	96.5

Becker M, *et al. Ann Rheum Dis* 2019; 0:1242–1248. doi: 10.1136/annrheumdis-2019-215145

Gastrointestinal	
Oesophageal symptoms	99.9
Stomach symptoms	99.3
Intestinal symptoms	99.3
Kidney	
Renal crisis	99.4
Skin	
mRSS	93.2
Worsening of skin changes within the last month	98.3
Worsening of finger vascularisation within the last month	98.3
Skin progression rate	88.2
Multiorgan	
VAI* >3	88.1

*VAI is a measure of disease activity in SSc.

To achieve a proportion of complete cases ≥25%, two predictors with the largest amount of missing data (body mass index and anti-RNA-polymerase III positive) were not considered for MI-LASSO.

ACA, anti-centromere antibody; ANA, anti-nuclear antibody; BMI, body mass index; CRP, C-reactive protein; DLCO, diffusion capacity of the lung for carbon monoxide; DU, digital ulcer; ESR, erythrocyte sedimentation rate; FEV_1, forced expiratory volume after 1 second; FVC, forced vital capacity; LVEF, left ventricular ejection fraction; MI-LASSO, multiple imputation – least absolute shrinkage and selection operator; mRSS, modified Rodnan skin score; TLC, total lung capacity; VAI, Valentini activity index.

Becker M, *et al. Ann Rheum Dis* 2019; 0:1242–1248. doi: 10.1136/annrheumdis-2019-215145

Supplementary table S2 Comparison of patients with and without missing data on the outcome

Demographics and baseline characteristics	Patients without missing data on outcome (n=706)	Patients with missing data on outcome (n=745)
Demographic		
Male sex	172 (24.4)	174 (23.4)
Age, years (mean±SD)	52.9±12.9	51.9±14.1
Disease duration, months (mean±SD)	101.1±94.0	94.5(±79.8
Bodyweight, mean±SD	64.6±13.4	67.3±15.3
Laboratory parameters		
ANA positive	657 (94.4)	631 (94.9)
ACA positive	48 (7.1)	58 (9.6)
Anti-Scl70 positive	414 (60.2)	372 (59.9)
Anti-U1RNP positive	27 (4.7)	25 (5.7)
CK elevation	64 (9.5)	68 (11.5)
Proteinuria	57 (8.4)	60 (10.2)
Hypocomplementaemia	39 (6.3)	38 (8.2)
ESR >20 mm/1h, mean±SD	25.3±20.6	24.5 (19.4)
CRP elevation	190 (27.7)	184 (30.7)
Vascular		

Becker M, *et al. Ann Rheum Dis* 2019; 0:1242–1248. doi: 10.1136/annrheumdis-2019-215145

Raynaud's present	683 (96.7)	703 (97.0)
DU	266 (38.1)	247 (35.8)
Active DU	126 (18.1)	123 (18.6)
Scleroderma (puffy fingers)	303 (44.2)	342 (53.2)
Musculoskeletal		
Tendon friction rubs	89 (12.8)	87 (12.8)
Joint synovitis	108 (15.4)	109 (15.9)
Joint contractures	310 (44.4)	323 (47.2)
Muscle weakness	164 (23.4)	167 (24.6)
Cardiopulmonary		
Pericardial effusion	58 (8.9)	39 (8.7)
Echocardiography suspected PH	113 (16.3)	73 (14.1)
Conduction blocks	104 (15.6)	73 (12.8)
Abnormal diastolic function	170 (25.0)	106 (21.0)
Lung fibrosis	131 (19.7)	75 (17.4)
Arterial hypertension	154 (21.9)	166 (23.9)
Dyspnoea	91 (13.2)	83 (14.1)
DLCO, %predicted (mean±SD)	64.1±20.2	63.1±19.7
FVC_1, %predicted (mean±SD)	86.4±21.3	86.4±20.9

Becker M, *et al. Ann Rheum Dis* 2019; 0:1242–1248. doi: 10.1136/annrheumdis-2019-215145

FEV_1, %predicted (mean±SD)	85.0±18.7	85.1±19.5
TLC, %predicted (mean±SD)	84.2±19.9	88.7±20.6
LVEF, %predicted (mean±SD)	61.7±7.0	60.4±8.3
Gastrointestinal		
Oesophageal symptoms	455 (64.5)	462 (64.1)
Stomach symptoms	192 (27.4)	176 (25.3)
Intestinal symptoms	177 (25.2)	208 (29.7)
Kidney		
Renal crisis	34 (4.8)	20 (2.9)
Skin		
mRSS, mean±SD	14.2±9.1	13.9±8.7
Worsening of skin changes within the last month	141 (20.3)	167 (24.5)
Worsening of finger vascularisation within the last month	162 (23.3)	187 (27.7)
Skin progression rate, mean±SD	0.6±1.6	0.5±1.4
Disease activity		
VAI* >3	191 (30.7)	177 (35.5)

Data are expressed as n (%) unless otherwise stated.

*VAI is a measure of disease activity in SSc.

Becker M, *et al. Ann Rheum Dis* 2019; 0:1242–1248. doi: 10.1136/annrheumdis-2019-215145

ANA, anti-nuclear antibody; ACA, anti-centromere antibody; CRP, C-reactive protein; ESR, erythrocyte sedimentation rate; DLCO, diffusion capacity of the lung for carbon monoxide; DU, digital ulcer; FEV_1, forced expiratory volume after 1 second; FVC, forced vital capacity; LVEF, left ventricular ejection fraction; MI-LASSO, multiple imputation – least absolute shrinkage and selection operator; mRSS, modified Rodnan skin score; PH, pulmonary hypertension; SSc, systemic sclerosis; TLC, total lung capacity; VAI, Valentini activity index.

Becker M, et al. Ann Rheum Dis 2019; 0:1242–1248. doi: 10.1136/annrheumdis-2019-215145

Supplementary table S3 Definition of Valentini activity index (VAI) for systemic sclerosis

Variable	Score
mRSS >14	1.0
Scleroderma (puffy fingers) = Yes / Current	0.5
Worsening of skin within the last month	2.0
Digital necrosis (gangrene)	0.5
Worsening of finger vascularisation within the last month	0.5
Joint synovitis	0.5
DLCO <80% predicted	0.5
Worsening of cardiopulmonary manifestations within the last month	2.0
ESR >30 mm/1h	1.5
Hypocomplementaemia	1.0
Maximum disease activity index score	10.0

DLCO, diffusion capacity of the lung for carbon monoxide; ESR, erythrocyte sedimentation rate; mRSS, modified Rodnan skin score.

Becker M, *et al. Ann Rheum Dis* 2019; 0:1242–1248. doi: 10.1136/annrheumdis-2019-215145

Supplementary table S4 Impact of predictors retained in the final model on long-term survival

Risk factor	Number of patients with data (of which had an event)	Median survival time (years)	95% CI (years)
Active DU*			
No active DU	645	4.48	3.70–4.97
Active DU	(245)	2.38	1.97–2.96
Corresponds to Figure S5A			
CRP elevation*			
No CRP elevation	765	4.48	3.7–4.97
CRP elevation	(365)	2.20	1.9–2.51
Corresponds to Figure S5B			
Lung fibrosis*			
No lung fibrosis	629	4.48	3.70–4.97
Lung fibrosis	(229)	2.00	1.74–2.32
Corresponds to figure S5C			
Muscle weakness*			
No muscle weakness	734	4.48	3.70–4.97
Muscle weakness	(334)	2.33	2.03–2.75

Becker M, *et al. Ann Rheum Dis* 2019; 0:1242–1248. doi: 10.1136/annrheumdis-2019-215145

Corresponds to			
figure S5D			
Active DU, CRP			
elevation, lung	411		
fibrosis and muscle	(11)		
weakness*		4.48	3.70–4.97
None of the above		1.13	0.32–1.74
All of the above			
Corresponds to			
figure S5E			
CRP elevation and			
lung fibrosis*			
None of the above	492	4.48	3.70–4.97
Both of the above	(92)	1.53	1.13–1.99
Corresponds to			
figure 2A			
CRP elevation and			
active DU*			
None of the above	493	4.48	3.70–4.97
Both of the above	(93)	1.82	1.23–2.47
Corresponds to			
figure 2B			

Becker M, *et al. Ann Rheum Dis* 2019; 0:1242–1248. doi: 10.1136/annrheumdis-2019-215145

For each risk factor (or a combination), the median survival time of patients with and without this risk factor (or combination thereof) have been calculated. The data correspond to figure 2 in the main text and online supplementary figure S5. *=p<0.001 by log-rank test.

CI, confidence interval; CRP, C-reactive protein; DU, digital ulcer

Becker M, *et al. Ann Rheum Dis* 2019; 0:1242–1248. doi: 10.1136/annrheumdis-2019-215145

Supplementary figure S1. Calibration curve of final model after bootstrapping

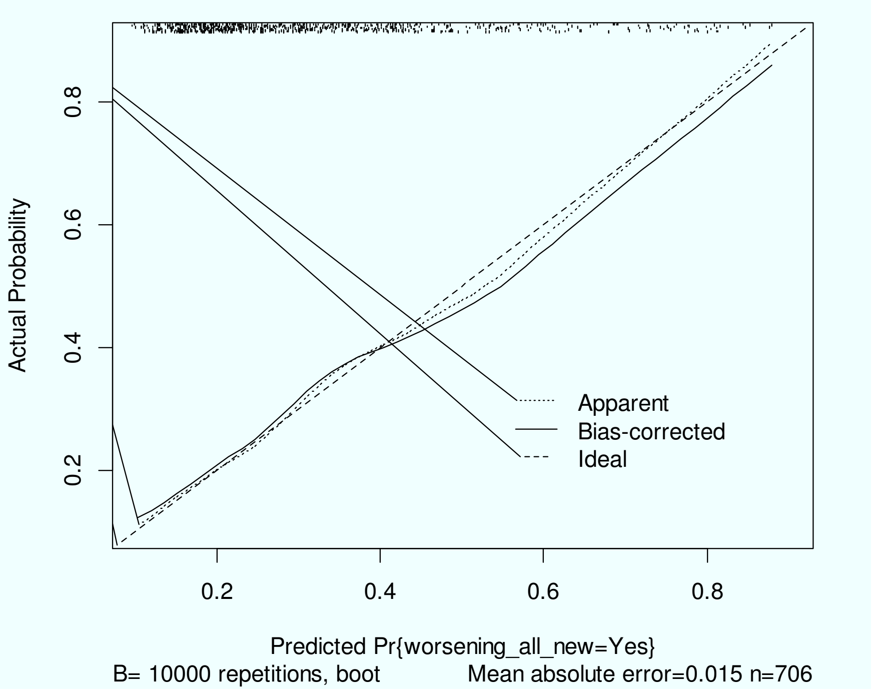

The plot shows observed responses against predicted responses. When a dataset is used to fit a regression model, the slope of observed versus predicted values is forced to be 1 (‘apparent’). However, when the model is applied to a different dataset, overfitting will cause the slope of the calibration plot to be <1 (‘bias-corrected’). For our model, the slope shrinkage factor (which is not to be confused with the shrinkage factor of the actual MI-LASSO regression analysis) was 0.926, and the maximum absolute error in predicted probability was only 0.026. Thus, the final model shows only minor overfitting.

Becker M, *et al. Ann Rheum Dis* 2019; 0:1242–1248. doi: 10.1136/annrheumdis-2019-215145

Supplementary figure S2 Scatterplot between FEV_1 and TLC (Pearson correlation, r=0.616)

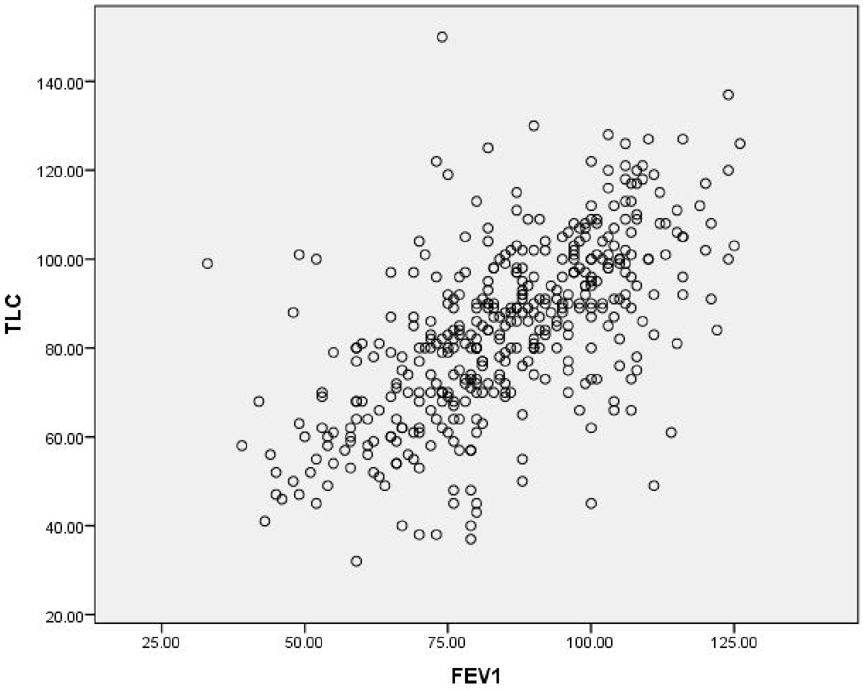

Becker M, *et al. Ann Rheum Dis* 2019; 0:1242–1248. doi: 10.1136/annrheumdis-2019-215145

Supplementary figure S3 Boxplot for FEV_1 stratified for patients with (n=139) and without (n=414) missing data on TLC. A two-samples t-test did not indicate a difference in means (p=0.510)

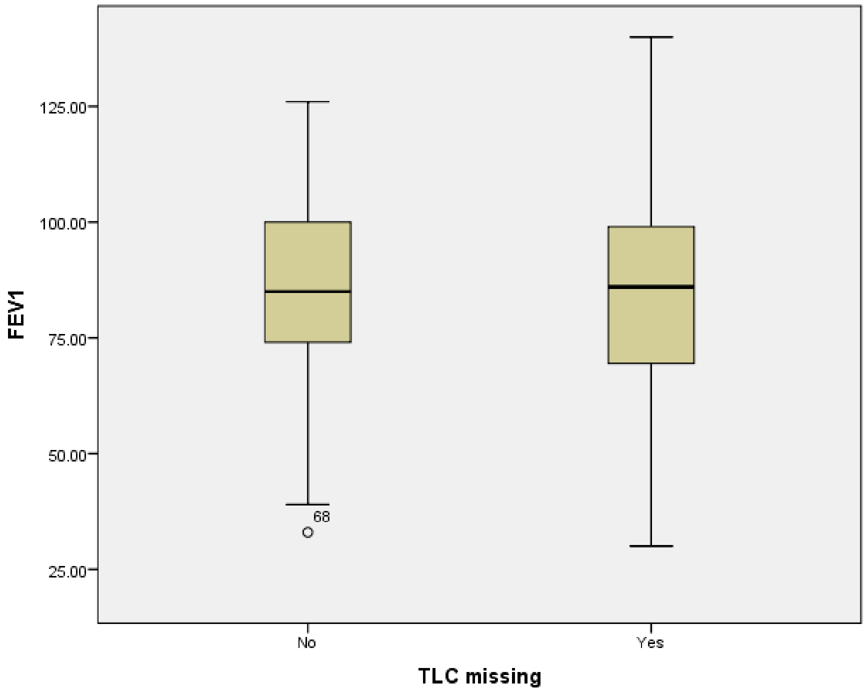

Becker M, *et al. Ann Rheum Dis* 2019; 0:1242–1248. doi: 10.1136/annrheumdis-2019-215145

Supplementary figure S4 Boxplot for TLC stratified for patients with (n=53) and without (n=414) missing data on FEV_1. A two-sample t-test did not indicate a difference in means (p=0.760)

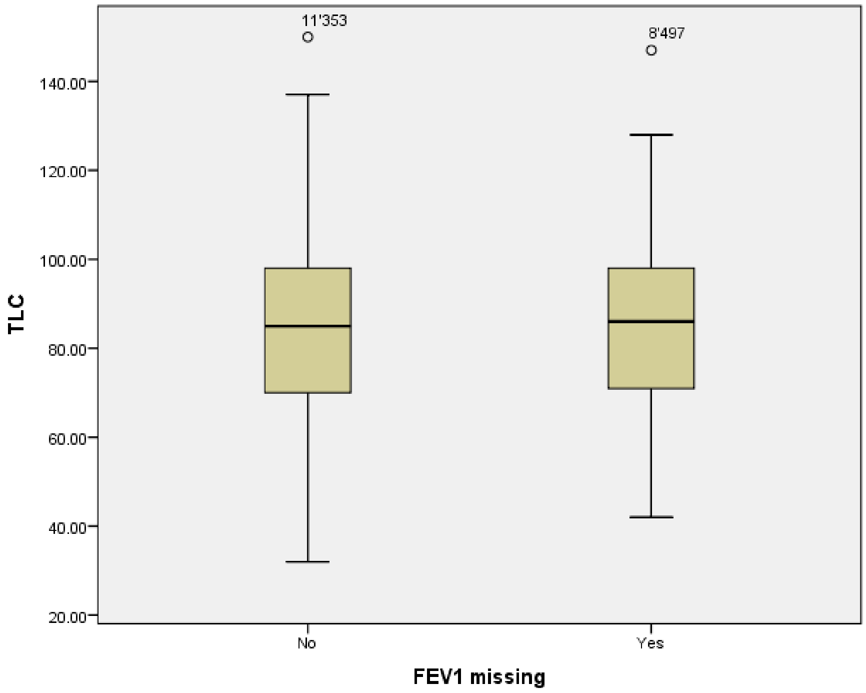

Becker M, *et al. Ann Rheum Dis* 2019; 0:1242–1248. doi: 10.1136/annrheumdis-2019-215145

Supplementary figure S5 Event-free survival of patients with SSc fulfilling the inclusion criteria (diffuse cutaneous SSc, death or at least one follow-up visit earliest at 12±3 months after baseline visit in 2009 or later) with risk factors (active DU – S5A; elevated CRP – S5B, presence of lung fibrosis – S5C, or muscle weakness – S5D; or their combination, S5E) versus no risk factors. The log-rank test was significant for all comparisons (p<0.001)

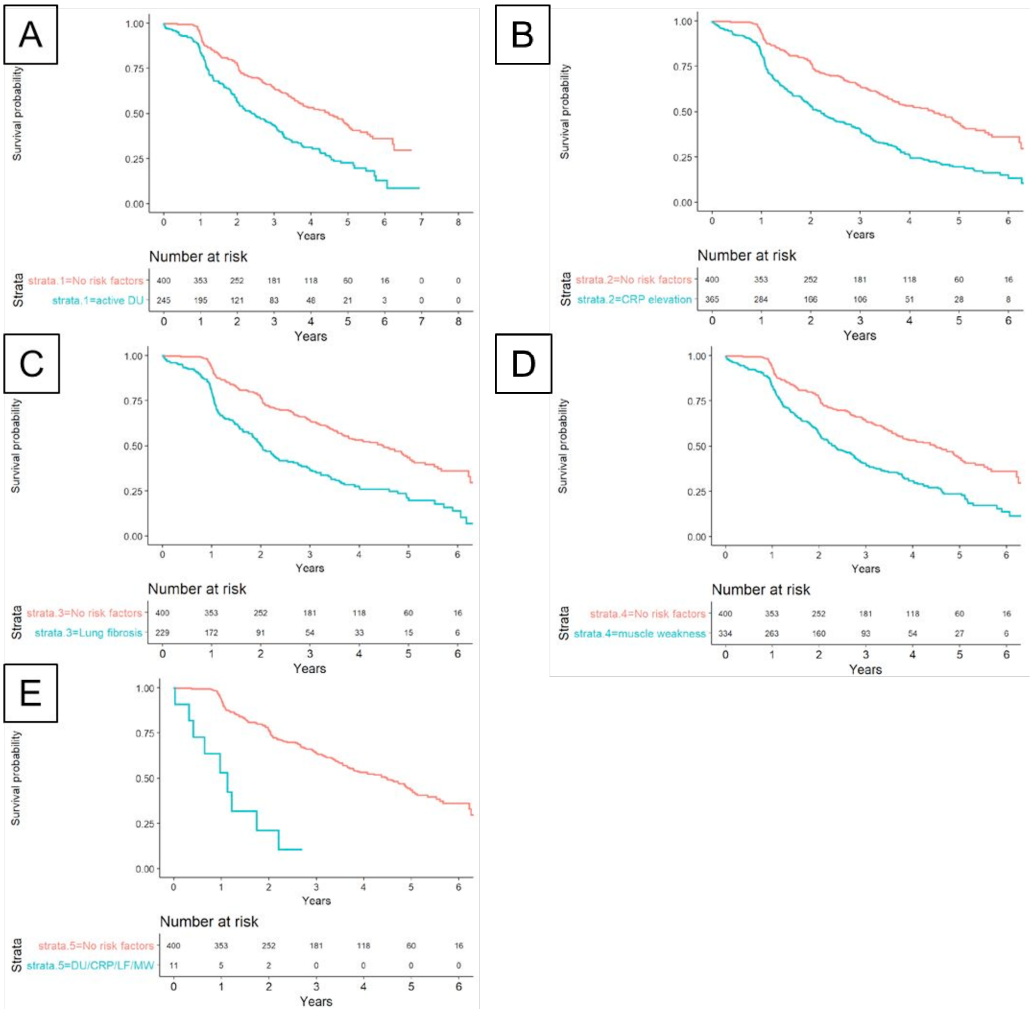

CRP, C-reactive protein; LF, lung fibrosis; DU, digital ulcer; MW, muscle weakness; SSc, systemic sclerosis.

Becker M, *et al. Ann Rheum Dis* 2019; 0:1242–1248. doi: 10.1136/annrheumdis-2019-215145
